# Three‐dimensional chromatin landscapes in somatotroph tumour

**DOI:** 10.1002/ctm2.1682

**Published:** 2024-05-20

**Authors:** Jing Guo, Yiyuan Chen, Haibo Zhu, Xinyu Tong, Lei Cao, Yazhuo Zhang, Weiyan Xie, Chuzhong Li

**Affiliations:** ^1^ Department of Neurosurgery Beijing Tiantan Hospital affiliated to Capital Medical University Beijing China; ^2^ Beijing Neurosurgical Institute Capital Medical University Beijing China; ^3^ Annoroad Gene Technology Co., Ltd Beijing China; ^4^ Beijing Institute for Brain Disorders Brain Tumor Center Beijing China; ^5^ China National Clinical Research Center for Neurological Diseases Beijing China

**Keywords:** 3D genome, Hi‐C, somatotroph tumour, TAD boundary

## Abstract

**Background:**

The three‐dimensional (3D) genome architecture plays a critical role inregulating gene expression. However, the specific alterations in thisarchitecture within somatotroph tumors and their implications for gene expression remain largely unexplored.

**Methods:**

We employed Hi‐C and RNA‐seq analyses to compare the 3D genomic structures of somatotroph tumors with normal pituitary tissue. This comprehensive approachenabled the characterization of A/B compartments, topologically associateddomains (TADs), and chromatin loops, integrating these with gene expression patterns.

**Results:**

We observed a decrease in both the frequency of chromosomal interactions andthe size of TADs in tumor tissue compared to normal tissue. Conversely, the number of TADs and chromatin loops was found to be increased in tumors. Integrated analysis of Hi‐C and RNA‐seq data demonstrated that changes inhigher‐order chromat in structure were associated with alterations in gene expression. Specifically, genes in A compartments showed higher density and increased expression relative to those in B compartments. Moreover, the weakand enhanced insulation boundaries were identified, and the associated genes were enriched in the Wnt/β‐Catenin signaling pathway. We identified the gainedand lost loops in tumor and integrated these differences with transcriptional changes to examine the functional relevance of the identified loops. Notably, we observed an enhanced insulation boundary and a greater number of loops in the TCF7L2 gene region within tumors, which was accompanied by an upregulation of TCF7L2 expression. Subsequently, TCF7L2 expression was confirmed through qRT‐PCR, and upregulated TCF7L2 prompted cell proliferation and growth hormone (GH) secretion in vitro.

**Conclusion:**

Our results provide comprehensive 3D chromatin architecture maps of somatotroph tumors and offer a valuable resource for furthering the understanding of the underlying biology and mechanisms of gene expression regulation.

## Graphical abstract

High‐resolution Hi‐C delineates the 3D chromatin landscape of somatotroph tumours.

Integrated Hi‐C and RNA‐seq analyses link chromatin structure changes to gene expression.

In the TCF7L2 gene area, we observed TADs gained and loops increased, along with an upregulation of its expression in the tumour, which potentially promotes tumour cell proliferation and GH secretion.

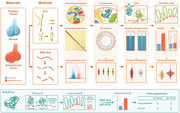

## 1 INTRODUCTION

The three‐dimensional genome organization has been shown to regulate gene transcription and play a crucial role in various cellular activities.^1^, ^2^, ^3^, ^4^, ^5^ Chromatin conformation capture techniques, most notably in situ Hi‐C, have been developed to determine genome architecture, by measuring the frequency of physical interactions between genomic regions.^6^ The mammalian genome is segregated into A and B compartments, which are associated with euchromatic (active gene expression) and heterochromatin (inactive gene expression) regions, respectively.^6^, ^7^ These compartments are composed of submegabase‐scale domains, called topologically associating domains (TADs), approximately 100 kb to 1 Mb in scale. High‐frequency interactions occur between genes and appropriate regulatory elements within TADs but not within other regions, which is important for transcription regulation and replication.^5^ Changes in TAD structures, such as size and boundaries, are related to abnormal gene regulation, developmental defects, and cancer.^8^, ^9^, ^10^, ^11^ Inside TADs, chromatin loops enable the interaction over long distances between enhancers and promoters, acting as structural units of gene regulation over distances ranging from 10 kilobases to 1 megabase.^2^, ^12^, ^13^ Furthermore, alterations in DNA loops have been implicated in a range of developmental abnormalities and human diseases.^10^, ^14^, ^15^

Pituitary neuroendocrine tumours (PitNETs), representing 10%−20% of intracranial tumours,^16^ are classified as either clinical functional or non‐functional, based on hormonal secretion status.^17^, ^18^ Approximately, 8%−15% of PitNETs secrete growth hormone (GH).^17^, ^19^, ^20^ The hypersecretion of GH usually causes gigantism in children or adolescents, while in adults, it leads to acromegaly and unfavourable metabolic changes and comorbidities, such as hypertension, insulin resistance, cardiovascular and respiratory dysfunction, and neoplastic complications.^21^, ^22^ The mechanisms and etiology of GH‐secreting PitNETs (somatotroph tumours) involve complex multistep processes that currently involve genetic and epigenetic alterations and the tumour microenvironment.^22^, ^23^, ^24^ Notably, recent findings by Franke et al. have demonstrated changes in the TAD structure that cause the misexpression of GPR101 in X‐linked acrogigantism.^25^ However, despite these advances, the comprehensive landscape of three‐dimensional (3D) genome architecture changes in somatotroph tumours, and the relationships between gene expressions have not yet been elucidated.

The Wnt/β‐catenin pathway plays a vital role in regulating the adhesion, invasion, and metastasis of pituitary tumour, and it significantly contributes to the development and oncogenesis of these tumours.^26^ TCF7L2 is a nuclear transcription factor (TF) that interacts with nuclear β‐catenin, facilitating the transcription of downstream Wnt target genes.^27^ Many studies have revealed that TCF7L2 facilitates disease development through interaction with the Wnt/β‐catenin pathway.^28^, ^29^ However, the structural alterations, expression profiles, and functional roles of TCF7L2 in somatotroph tumours have not been fully elucidated.

Herein, high‐resolution in situ Hi‐C and RNA sequencing were performed to determine the chromosome structure and gene expression profile of pituitary normal tissue and somatotroph tumours. Through the combined analysis of Hi‐C and RNA‐seq data, we discerned a correlation between alterations in chromatin structure and gene expression variations. Our research advances knowledge of the 3D chromatin landscape's impact on the genesis and progression of somatotroph tumours.

## 2 MATERIALS AND METHODS

### 2.1 Primary sample collection

Tumour samples were obtained from patient who was diagnosed with somatotroph tumour and underwent surgical resection at Beijing Tiantan Hospital. No patients had undergone radiotherapy or chemotherapy prior to surgery. Normal pituitary tissue was collected from healthy donors. Information on the samples is provided in Table S1. The study received ethical approval from the Ethics Committee of Beijing Tiantan Hospital, and written informed consent was obtained from all participants before any procedures were conducted.

### 2.2 Hi‐C library preparation and data analysis

In situ Hi‐C experiments of tumour and normal pituitary tissues were performed following a previously described protocol.^13^ In situ Hi‐C was performed in triplicate for solid somatotroph tumour (tumour library 1, 2 and 3), in duplicate for normal pituitary gland 1 (normal library 1 and 2), and in triplicate for normal pituitary gland 2 (normal library 4, 5 and 6, Figure S1). Briefly, samples were crosslinked with 2% formaldehyde and quenched with 2.5 M glycine. The crosslinked tissue was lysed, incubated on ice and centrifuged to pellet nuclei. Nuclei were washed and resuspended in restriction enzyme buffer, then chromatin was solubilised with dilute SDS and quenched with Triton X‐100. Genome digestion was performed overnight using MboI restriction enzyme. After digestion, DNA ends were biotin‐ labelled and blunt‐end ligated. The nuclear complexes were de‐crosslinked, and DNA was purified and sheared. After end repair, biotin enrichment, and A‐tailing, Hi‐C libraries were prepared. Libraries were PCR amplified and sequenced on Illumina HiSeq platform (Supporting information).

Raw reads from the Hi‐C data were processed by Hi‐C Pro^30^ to create a raw contact count matrix, and mapped to the human genome hg19 by Bowtie2 (v2.3.4).^31^ The iterative correction method was used to normalise the matrix. The HiCRep^32^ package provides stratum‐adjusted correlation coefficients to assess the reproducibility and quality of Hi‐C libraries. Interaction decay exponents (IDE)^6^, ^33^ values were calculated for each chromosome to describe the trend of interaction frequency with distance.

### 2.3 RNA‐seq and data analysis

Total RNA was isolated and purified from the frozen tissue through the miRNeasy Mini Kit (QIAGEN, #217004), adhering to the protocol provided by the manufacturer. RNA sequencing was performed on the same samples used for in situ Hi‐C, including triplicates for the solid somatotroph tumour, duplicates for normal pituitary 1, and a single library for normal pituitary 2. Concisely, each RNA sample preparation utilised 2 μg of RNA as input. Libraries for sequencing were prepared with the NEBNext Ultra RNA Library Prep Kit for Illumina (#E7530L, NEB). Library quality was assessed with the Agilent Bioanalyzer 2100 system (Agilent). Sequencing was then carried out on an Illumina platform, producing 150 bp paired reads (Supporting information).

The sequences were aligned to the hg19 sequence (GRCh37) using HISAT2.^34^ The resulting binary alignment map (BAM) files were converted to bigwig format using deepTools bamCoverage, and the RNA transcript signals were visualised at 10 kb resolution. Gene read counts were obtained using HTseq,^35^ and DESeq2^36^ was employed to conduct differential expression analysis. kyoto encyclopedia of genes and genomes (KEGG) pathway analysis was conducted utilising the gseapy Python package (https://gseapy.readthedocs.io/en/latest/).

### 2.4 Identification of A and B compartments

Cworld‐Dekker software (Dekker Laboratory, https://github.com/dekkerlab/cworld‐dekker) was used to detect A/B compartments at 100 kb resolution.

### 2.5 Topologically associated domains boundary analysis

We executed insulation analysis and TAD calling on 10 kb resolution cooler matrices, employing the insulation utility of cooltools (https://github.com/open2c/cooltools). After the tumour and normal tissues were called for insulation boundaries, we identified high‐confidence boundaries by excluding positions where the log2 insulation score was greater than 0 in both groups. We defined the difference boundaries by the absolute value of the difference in the log2 insulation score > .4. Motif enrichment analysis of enhanced insulation boundary sequence datasets was performed with meme (https://meme‐suite.org/meme/tools/sea).

### 2.6 Chromatin loop analysis

Hi‐C Computational Unbiased Peak Search (HiCCUPS) was used to identify genome‐wide chromatin loops at 10 kb bins, and the default parameters were used.^37^ HiCCUPSDiff was used to check the loops called in the tumour and normal hic files, after which the tumour gain and lost loops were determined by the observed/expected ratio. Then, a comparison was made. Aggregates of specific loops were plotted by Coolpup.py v.1.0.0^38^ at 10 kb resolution.

### 2.7 Cell culture and transfection

The GH3 (CCL‐82.1) rat pituitary cell line was acquired from the American Type Culture Collection (ATCC). The cells were cultured in Ham's F12K medium supplemented with 2.5% fetal bovine serum (FBS) and 15% horse bovine serum (Gibco). The cultures were maintained in a humidified incubator at 37°C with 5% (v/v) CO_2_ and the media were changed after every 2 days. We measured GH levels in the cell culture supernatant utilising a Rat Growth Hormone ELISA Kit (SenBeiJia Biological Technology Co., Ltd., Nanjing, China) as described by the manufacturer.

A short hairpin RNA (shRNA) plasmid targeting rat Tcf7l2 and nontargeting shRNA (sh‐NC) were obtained from WZ Biosciences (Shandong, China). The shRNA sequences used were as follows: Tcf7l2 shRNA1: 5′‐GCAGTGCCGTTTCTTTCTTTCTTCAAGAGAGAAAGAAAGAAACGGCACTGCTTTTTT‐3′; shRNA2: 5′‐CCCTCCAGATATCTCTCCATATTCAAGAGATATGGAGAGATATCTGGAGGGTTTTTT‐3′. The Tcf7l2 overexpression plasmid, pcDNA3.1‐FLAG‐Tcf7l2, was constructed by BAC Biological Technology Co., Ltd. (Beijing, China). Transfections, including shRNAs and overexpression plasmids, were executed with Lipofectamine 3000 (Invitrogen, USA). Briefly, GH3 cells (1 × 10^6^ per well) were plated in 6‐well plates 1 day before transfection. The plasmids (2.5 μg) were diluted in 125 μL of Opti‐MEM, mixed with 10 μL of P3000 reagent, and combined with 7.5 μL of Lipofectamine 3000. After 10 min of incubation, the mixture was added dropwise to the cells. After 6 h, the medium was changed, and the cells underwent further incubation for 24−48 h for subsequent experiments.

### 2.8 qRT‐PCR and western blot analysis

The experimental procedures used for qRT‐PCR and Western blotting were described in our previous publication.^39^ Briefly, we extracted total RNA using the RNeasy Minikit (QIAGEN, #74104), and synthesised cDNA using Thermo Fisher Scientific's High Capacity cDNA Reverse Transcription Kit. We conducted qRT‐PCR using Thermo Fisher Scientific's Power SYBR Green PCR Master Mix, and analysed the results with QuantStudio 3 and 5 systems from Applied Biosystems. Gene expression levels were determined through the comparative Ct (cycle threshold) method, utilising GAPDH as the housekeeping gene. The sequences of primer used were as follows: Tcf7l2‐rat forward 5′‐CGCTAACGACGAACTGATCTCC‐3′ and reverse 5′‐ ATTCATTGACCAACGACGACT‐3′; TCF7L2‐homo forward 5′‐TCGTAGCTGAGTGCACGTTG‐3′ and reverse 5′‐CGGGCCAGCTCGTAGTATTT‐3′.

During Western blot analysis, we extracted total protein using NCM Biotech's RIPA buffer and determined its concentration through Thermo Fisher Scientific's BCA assay. Equal protein amounts were separated by sodium dodecyl sulfate polyacrylamide gel electrophoresis (SDS‐PAGE) and then transferred onto a polyvinylidene difluoride (PVDF) membrane. Primary antibodies, including TCF7L2 (1:2000, Abcam, ab272235) and GAPDH (1:6000, Abcam, ab8245), were used for target protein detection. The protein bands were visualised using an Amersham Imager 600 (GE Healthcare). GAPDH served as the loading control for normalization.

### 2.9 Cell counting kit‐8 proliferation assay

Cell viability was evaluated using a Cell counting kit‐8 (CCK‐8) (Dojindo, Japan). GH3 cells post‐transfection were plated in 96‐well plates at 3 × 10^4^ cells per well. At various time points (24, 48, 72 and 96 h), we added 10 μL of CCK‐8 reagent to each well and measured the absorbance at 450 nm.

### 2.10 Statistical analysis

Statistical computations were conducted with R v3.4.1 (https://www.r‐project.org/), Python 3.10.8, and Prism 8 (GraphPad Software, Inc.). Statistical significance was determined by unpaired Student's *t*‐test (two groups) or one‐way ANOVA (multiple groups). *P*  <  .05 was considered to indicate statistical significance. Multiomics display figures were generated using Trackc (https://github.com/seqyuan/trackc).

## 3 RESULTS

### 3.1 Alterations in the genome‐wide interaction matrix of somatotroph tumour

**FIGURE 1:**
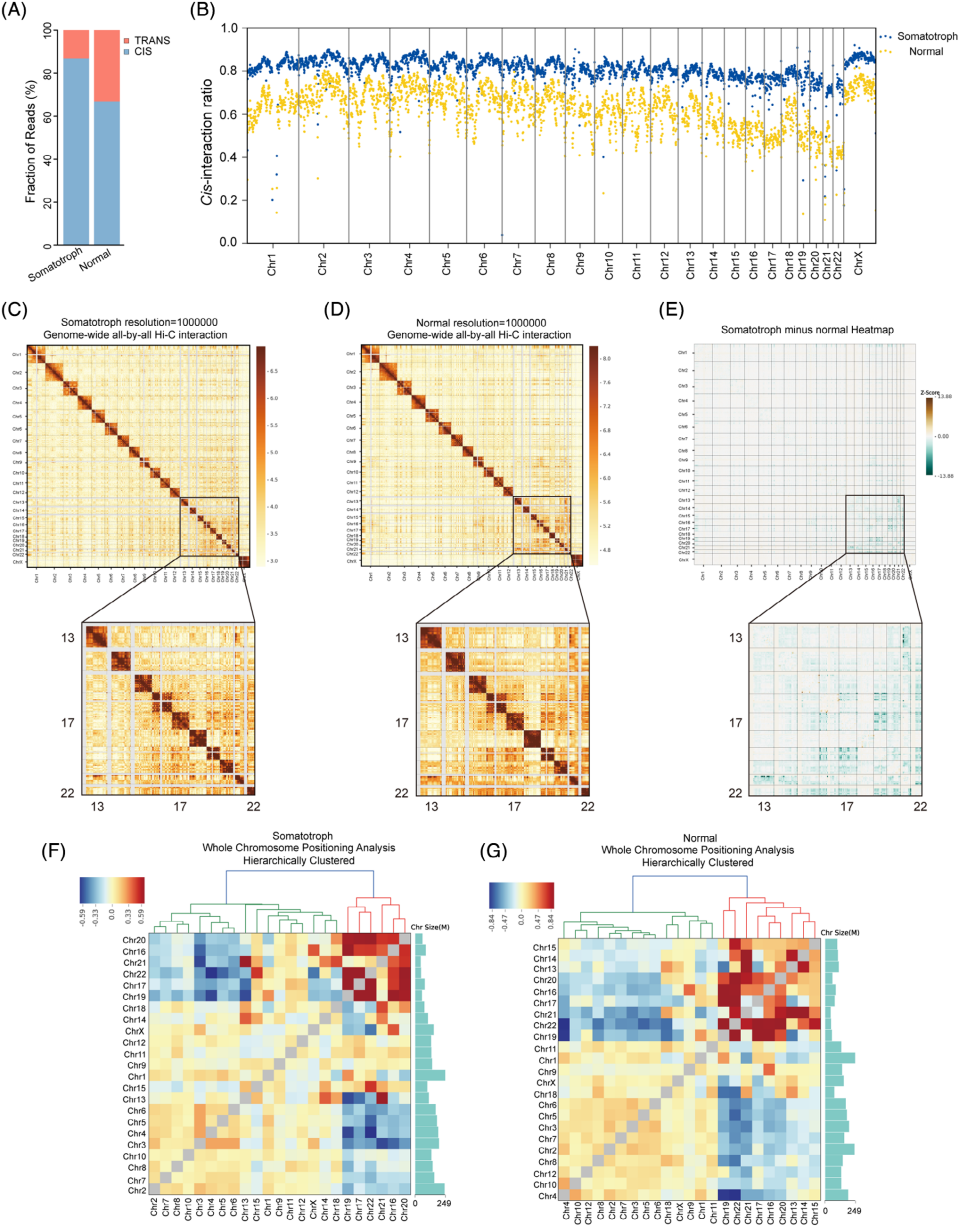
Alterations in the genome‐wide interaction matrix of somatotroph tumour. (A) Read fraction of inter‐chromosomes (*trans*) and intra‐chromosome (*cis*) interactions. (B) *Cis*‐interaction proportion along each chromosome between tumour and normal pituitary. (C,D) Genome‐wide all‐by‐all Hi‐C interaction map at 1 Mb resolution in tumour (C) and normal pituitary (D). The chromosomes are stacked in numerical order from left to right and top to bottom. The colour bar depicts the frequency of Hi‐C interactions. In the lower panels, enlargements of the *cis*‐ and *trans*‐interactions for chr13 through chr22 are shown. (E) Genome‐wide heatmap of relative contact differences between tumour and normal pituitary tissue. The relative difference is calculated as the log2 ratios of the Hi‐C matrices of the tumour and normal pituitary tissue. The lower panel represents the enlargement of the chr13 through chr22 region. (F,G) Inter‐chromosomal interactions between all pairs of chromosomes in tumour (F) and normal pituitary tissue (G). Each block represents observed/expected interactions between chromosomes. Red and blue indicate enrich and depleted, respectively. The histogram on the right shows the chromosome size; the coordinates are in M units.

To explore 3D chromatin organization in somatotroph tumour, we performed in situ Hi‐C sequencing of somatotroph tumour and normal pituitary glands to generate high‐resolution Hi‐C maps. After sequencing filtering, a total of 2.7 billion valid paired‐end reads were obtained from the Hi‐C data (Table S2). The biological replicates of each Hi‐C experiment were highly reproducible (Figure S1). Over 80% of bins exhibited a depth exceeding 1 000 at a 5 kb resolution (Figure S2A,B), indicating that our interaction map was constructed at a resolution of 5 kb. In addition, we investigated the proportion of intra‐chromosomal (‘*cis*’) and inter‐chromosomal (‘*trans*’) interactions in both tumour and normal pituitary tissues. By analysing the correlation and the *cis*/*trans* ratio across different libraries from different samples, we observed a substantial similarity in the 3D chromatin organisation between normal pituitary glands from two different donors. This similarity establishes a baseline for comparison with the tumour samples (Figures S1, S3A, and S3B). Our findings indicate a higher prevalence of *cis* interaction compared to *trans* interaction (Figure 1A), and that the proportion of *cis*‐interaction in tumour was greater than that in normal tissues (Figures 1A,B and S3C).

To further investigate the chromosome interactions, we constructed genome‐wide matrix maps, in which darker red represents more frequent interaction events (Figure 1C,D). In agreement with chromosome territories concept,^40^ we observed that intra‐chromosomal interactions, appearing as darker boxes along the diagonal, occurred more frequently than inter‐chromosomal interactions (Figure 1C,D). Circos^41^ analysis revealed the top 1 000 inter‐chromosome interactions at 1‐Mb resolution in tumour and normal pituitary tissue (Figure S4A–C). Compared to those in the normal pituitary gland, the strength of pairwise genome‐wide chromatin interactions in somatotroph tumour was decreased, especially for small chromosomes (chr16–22), as evidenced by matrix subtraction between normal tissue and tumour Hi‐C contact maps (Figure 1E). For each chromosome, the frequency of chromatin interactions decreased within a relatively close range of chromosome distances (Figure S5A,B). The occurrence of intra‐chromosomal interactions diminished as the linear distance increased (Figure S5C).

Furthermore, the correlations between inter‐chromosomal interactions, chromosome size and spatial position were further explored. Our findings indicate that smaller, gene‐dense chromosomes (chr16, 17, 19, 20, 21, 22) are more likely to interact with each other, demonstrating that proximity markedly influences contact probability (Figure 1F,G), consistent with earlier studies.^6^, ^42^

### 3.2 A/B compartment flipping in somatotroph tumour

**FIGURE 2:**
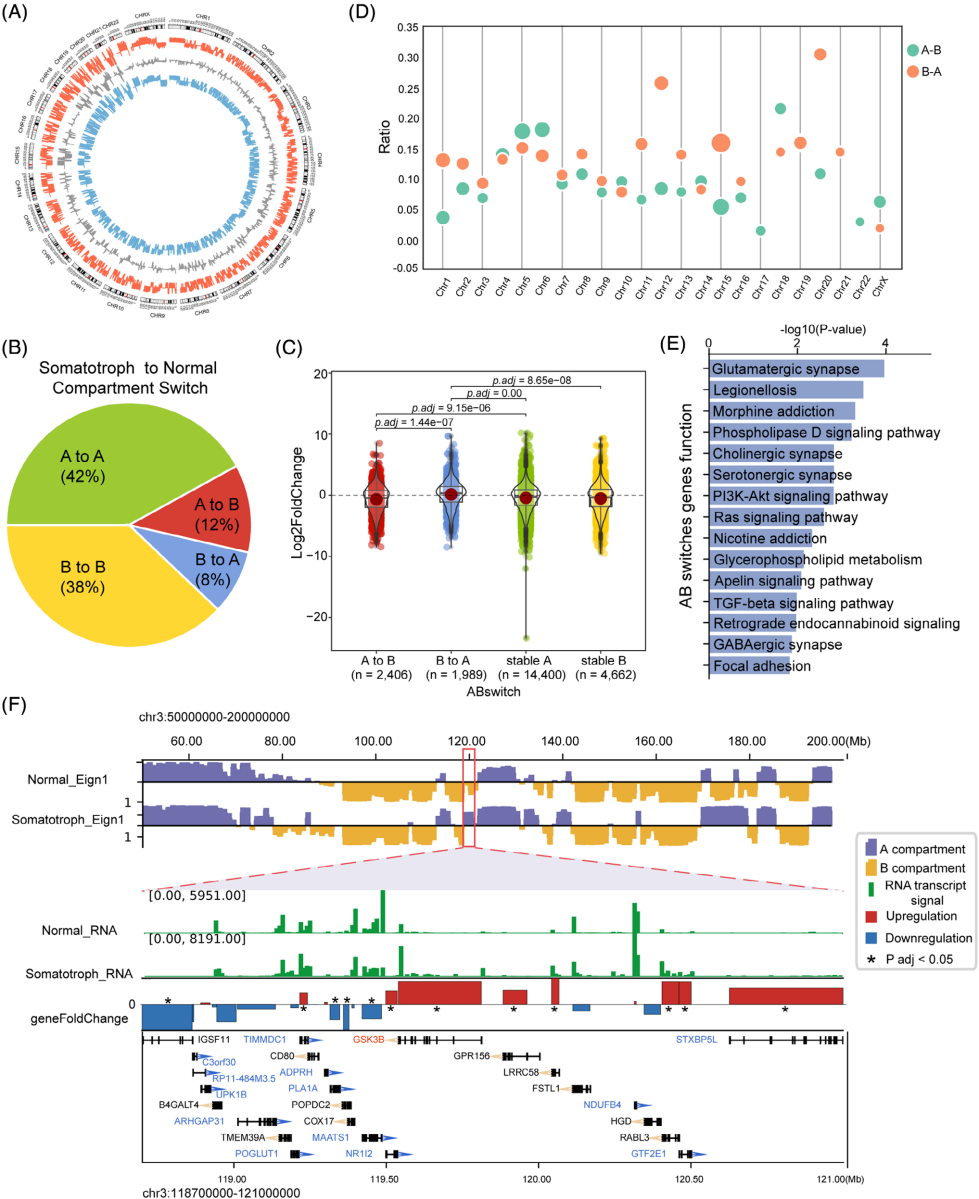
A/B compartment flipping in somatotroph tumour at 100 kb resolution. (A) Genome‐wide landscape of switched compartments from tumour to normal pituitary. From outside to inside, circles show each chromosome, eigen value of the first principal component (PC1) from tumour, overlap of eigen value of PC1, and eigen value of PC1 from normal pituitary. (B) The percentage of stable (A‐to‐A or B‐to‐B) or switched compartments (A‐to‐B or B‐to‐A) between tumour and normal pituitary in genome. (C) Box plots showing the comparison of gene expression levels in different compartments between tumour and normal pituitary. Gene expression was compared as Log2FC (tumour/normal pituitary) with the *p*‐value obtained by the Wilcoxon rank‐sum test. (D) Bubble diagram showing percentage of differentially expressed genes (DEGs) moving between compartments. Size of bubbles indicates the number of genes. (E) KEGG enrichment analysis of DEGs in the A/B compartments switched region. (F) The representative genomic region 40−200Mb on chr3 of somatotroph tumour and normal tissue displayed A/B compartments. The top two lanes indicated the first principal component values corresponding to A compartments and B compartments in normal and tumour, respectively. The third and fourth lanes indicated the RNA transcript signal of genes in normal and tumour, respectively. The fifth lane indicated the DEGs between normal and tumour. Red bars represented the upregulation, and the blue bars represented the downregulation. For example, GSK3B, located in the B‐to‐A compartment, is significantly upregulated in somatotroph tumour.

To explore the relationship between genomic structural changes and transcriptional levels, we performed RNA sequencing on the same samples used for in situ Hi‐C. Based on RNA sequencing data, a total of 22 202 genes were identified, with 4395 genes upregulated and 5667 genes downregulated (|Fold change| > 1, *P*_adj_ < .05, Figure S6). We used a 100‐kb Hi‐C matrix to divide the chromatin of tumour and normal pituitary tissues into A and B compartments (Figures 2A, S7A; Table S3), representing active and inactive chromatin regions, respectively. Our findings indicate that compartmentalization was predominantly consistent between tumour and normal tissues, comprising 42% constitutive A compartments and 38% constitutive B compartments of the genome (Figures 2B and S7B). Compared to normal pituitary tissue, our study identified that 20% of the genomic compartments in tumours had switched states, with a higher transition from A to B (12%) compared to B to A (8%) (Figure 2B). Additional analysis incorporating RNA‐seq data revealed a significant correlation between compartment statuses (A, B, and switching) and gene expression changes (Table S4). Our analysis of all genes identified by RNA‐seq demonstrated that both gene expression levels and gene density were significantly elevated in stable A‐type and ‘B to A’ switched compartments compared to stable B‐type and ‘A to B’ switched compartments (Figure 2C, Table S4). Furthermore, by calculating the ratio of differentially expressed genes (DEGs) to the total genes shifting compartments on each chromosome, we found chromosome 20 exhibited the highest fraction of genes transitioning from B to A, and chromosome 18 had the most genes shifting from A to B (Figure 2D).

To explore the function of the genes associated with AB switching, we performed enrichment analysis on significant DEGs (|Fold change| > 1, *p*_adj_ < .05) associated with AB transitions. The results showed that these genes were mainly enriched in Glutamatergic synapse, PI3K‐Akt signalling pathway, and TGF‐beta signalling pathway (Figure 2E, Table S5). We further investigate the set of genes undergoing A/B switches within the PI3K‐Akt signalling pathway, known to be active in somatotroph tumour.^43^ Our results indicated that GSK3B, a key effector within the PI3K‐Akt pathway, underwent a switch from compartment B to A and was upregulated in somatotroph tumour (Figure 2F, Log2FC 2.22, *p*_adj_ < .001; Table S4).

Collectively, our data show that the chromatin A/B compartment's spatial distribution is distinct in tumours relative to normal tissues, with these alterations markedly linked to the expression changes of genes involved in cancer‐related pathways.

### 3.3 Topologically associated domain alterations in somatotroph tumour

**FIGURE 3:**
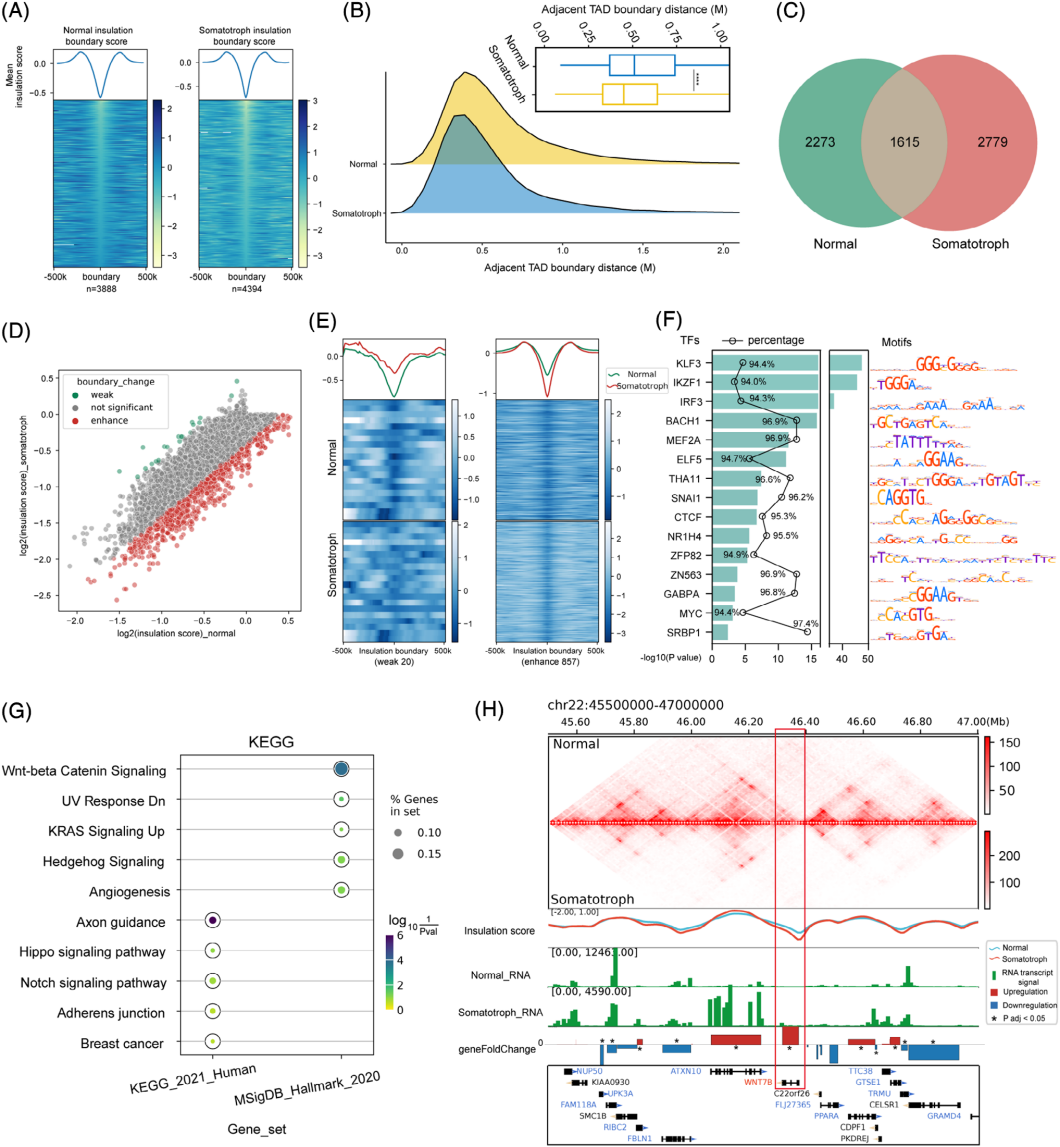
TAD alterations in somatotroph tumour at 10 kb resolution. (A) Histograms showing the number of TADs from HiCDB interaction matrices at 10‐kb resolution in the normal and tumour sample. (B) Adjacent TAD boundary distance comparison of all the TADs in normal and tumour sample. Ridge plots show the adjacent TAD boundary distance distribution. Bar plots show statistics (****P  <  .0001). (C) Venn diagram of TAD boundaries identified in tumour and normal tissue. (D) Scatter plot comparing insulation score in tumour and normal tissue Hi‐C samples. The green dot indicates that the boundary strength of the tumour is weakened compared to normal tissue, while the red dot is enhanced. (E) Average insulation score profiles of tumour and normal tissue at weaker and enhanced insulation boundaries. (F) Top 15 known TF motifs enriched at enhanced boundaries. The *p*‐values for motif enrichment were calculated by the HOMER software (v4.10), and a *p*‐value < .01 was considered to be statistically significant by default. The percentages of target sequences with TF motifs are indicated by the black polygonal chain. (G) The functional enrichment analysis of the enhanced insulation boundary related genes. (H) Hi‐C contact map of the 45.4–47.0 Mb region of chromosome 22. The upper and lower heat maps represented normal and tumour, respectively. The insulation score and RNA transcript signal comparison between normal and tumour showed below. A red frame denotes a common TAD boundary in both normal and tumour covering the WNT7B gene. TAD, Topologically associated domain.

TADs are the functional units of the chromatin architecture.^44^ To investigate alterations in TAD structures between tumour and normal tissues, we defined TADs in the samples at a 10 kb resolution employing insulation score algorithms. A total of 3 888 and 4 394 TAD boundaries were identified in normal tissues and tumour, with adjacent TAD boundary distances averaging 653 and 576 kb, respectively (Figure 3A,B). As expected, the insulation scores of the insulation boundaries were lower than those of the flanks (Figure 3A), indicating the reliability of the identified TAD boundaries. A comparison of TADs revealed that approximately 37% (1 615) of the TAD boundaries were conserved within tumour, and 63% (2 779) of the TADs were specific (Figure 3C, Table S6).

We then compared the boundary insulation score of each TAD between tumour tissue and normal tissue. We found that the boundary strengths of 20 TADs in tumours were weaker than those in normal tissues, while the boundary strengths of 857 TADs were stronger (Figure 3D,E, Table S6). To further identify TFs that are potentially involved in enhanced boundaries in tumour, we utilised HOMER software (v4.10)^45^ to analyse enhanced TAD boundary sequences and identify significantly enriched TF motifs. Within the enhanced boundaries, we found an enrichment of binding motifs including KLF3, IKZF1, and, as expected, the CTCF protein (Figure 3F).

To further explore the impacts of boundary changes at the transcriptomic level, we analysed gene expression under weak and enhanced insulation boundaries. We identified 434 genes related to the enhanced insulation boundary and 11 related to the weak insulation boundary. The results showed that the expression of the genes related to the enhanced boundary was greater than that of the genes related to the weak boundary (Figure S8A, Table S7). To determine transcriptome‐level alternations at the enhanced insulation boundary, we discerned 118 genes upregulated and 89 genes downregulated in tumour with *P*_adj_ < .05 and |Log2FC| > 1 (Figure S8B, Table S7). Functional enrichment analysis showed that these genes predominantly participated in pathways like Wnt‐beta Catenin, Hippo and Notch signalling (Figure 3G, Table S8). To investigate topological alterations in critical Wnt pathway genes, we analysed genes associated with enhanced and weak insulation boundary regions. Our analysis revealed significant structural and expressional changes in WNT7B, which is essential for the Wnt signalling pathway.^46^ Notably, we observed that insulation boundary of WNT7B was enhanced in tumour compared to normal tissue, correlating with an upregulation in its expression levels in tumour samples (Figure 3H, Table S7).

### 3.4 Chromatin loop alterations in somatotroph tumour

**FIGURE 4:**
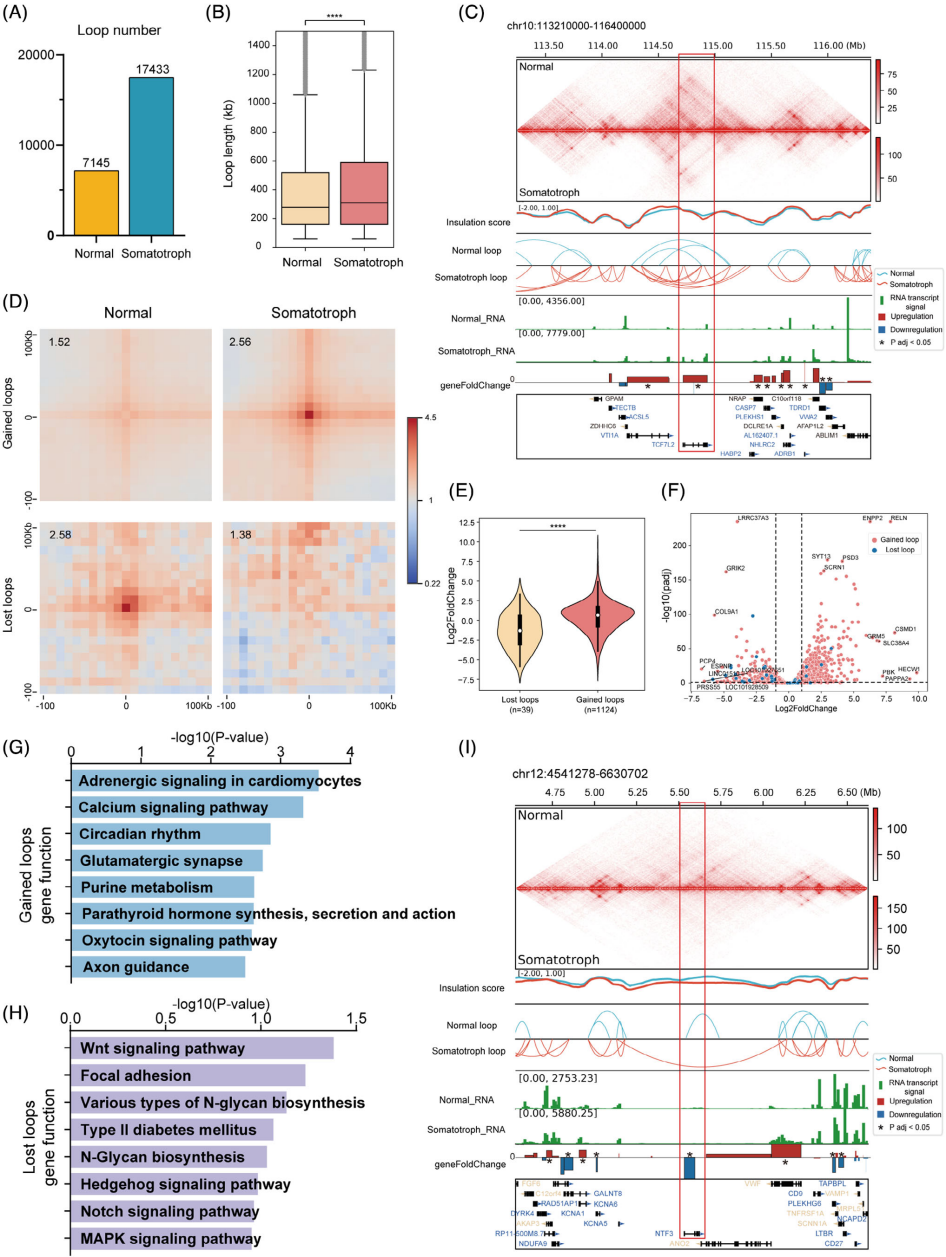
Chromatin loop alterations in somatotroph tumour at 10 kb resolution. (A) Box plot showing the number of loops and (B) bar plot showing the size of loops in the tumour and normal tissue. (C) Hi‐C contact map of the 113.0–116.5 Mb region of chromosome 10, along with insulation score, loop comparison, RNA transcript signal, and foldchange of genes. A red frame denotes a TAD boundary and loop alternation in normal and tumour covering the TCF7L2 gene. (D) Aggregate peak analysis (APA) plots measuring the aggregate strength of the tumour‐specific (*n* = 1357) and normal‐specific loops (*n* = 36). APA score was calculated with HiCPeaks software using Hi‐C matrix data at 5‐kb resolution. (E) Violin plots showing the comparison of gene expression levels in gain and lost loops. (F) Volcano plot showing the DEGs associated with gain and lost loops. The functional enrichment analysis of the gained loops related genes (G) and lost loops related genes (H). (I) Hi‐C contact map of the 4.50–6.72 Mb region of chromosome 12, along with insulation score, loop comparison, RNA transcript signal and foldchange of genes. A red frame denotes a TAD boundary and loop alternation in normal and tumour covering the NTF3 gene. TAD, Topologically associated domain; DEG, differentially expressed gene.

Chromatin loops facilitate the connection of distal *cis*‐regulatory elements (CRes), including enhancers, with their target genes, playing crucial roles in the regulation of the gene expression.^47^ To explore chromatin loop alternation, we identified 7 145 loops in normal tissue and 17433 loops in tumour using 10‐kb resolution matrices (Figure 4A). In addition, we found that the loop size of the tumour was larger than that of the normal tissue (Figure 4B). We investigated the loop alternation of TCF7L2, a critical TCF/LEF family TF implicated in the Wnt pathway.^27^ The results showed that the number of tumour loops associated with TCF7L2 was greater than that associated with normal tissue, and the expression level was up‐regulated in tumour tissue (Figure 4C). To explore the specific looping events in tumour, we identified 1 124 gained loops and 39 lost loops in the tumour sample (Table S9). Aggregate peak analysis (APA) plots were generated to evaluate the strength of the aggregates of the loops, which indicated that the gained and lost loops differed significantly in terms of the frequency of contact between the tumour and normal tissue (Figure 4D). To further investigate the relationship between loop alternations and differential gene expression, we identified 1 124 and 39 DEGs that were associated with the tumour gained and lost loops, respectively (Figure 4E,F, Table S9). We found that the gained loops were significantly more correlated with upregulated gene expression compared with lost loops (Figure 4E). The functional enrichment analysis of these genes revealed that genes associated with gained loops are involved in the calcium signalling pathway (such as, RYR1, CHRM3 and PDE1C) and purine metabolism (Figure 4G, Table S10). The genes associated with the lost loops were involved in pathways including Wnt signalling, focal adhesion, Notch signalling and MAPK signalling (Figure 4H, Table S11). To investigate loop alterations in critical genes of the MAPK pathway, we focused on genes associated with lost loops. Our analysis identified significant structural and expressional changes in Neurotrophin 3 (NTF3), known as a tumour suppressor in hepatocellular carcinoma that promotes apoptosis through the MAPK pathway.^48^ Specifically, we observed a loss of loops in the NTF3 gene region in tumour, accompanied by a significant downregulation of the NTF3 expression compared to normal tissue (Figure 4I).

### 3.5 TCF7L2 is upregulated in somatotroph tumours and promotes cell proliferation and growth hormone secretion

**FIGURE 5:**
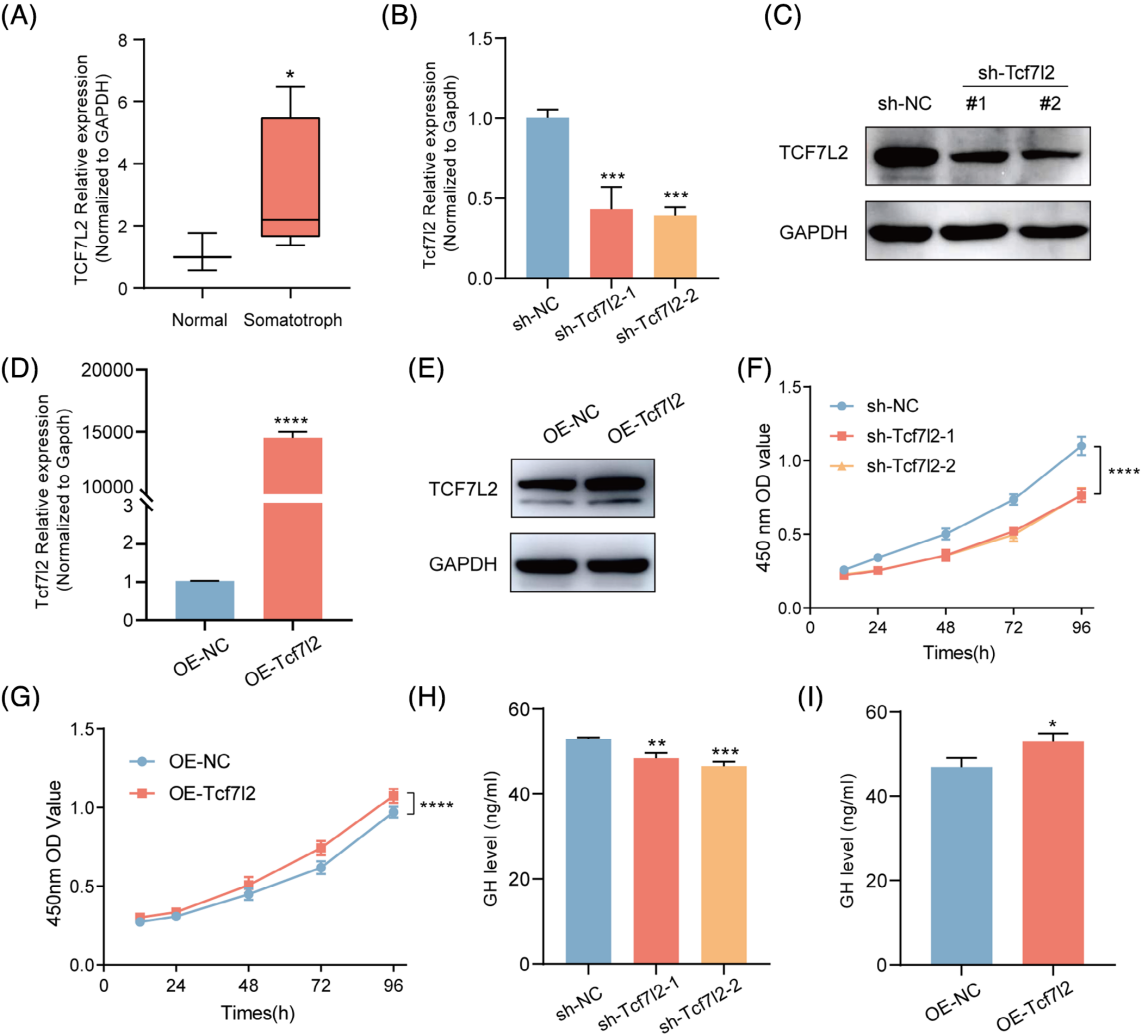
TCF7L2 is upregulated in somatotroph tumour and promotes cell proliferation and GH secretion. (A) TCF7L2 mRNA expression in normal pituitary (*n* = 3) vs. somatotroph tumour (*n* = 10). The results are expressed as mean  ±  SD (Student's *t*‐test, *P  <  .05). (B) Quantitative PCR and western blot (C) analysis of TCF7L2 knockdown efficiency in GH3 cells. *P*‐values by one‐way ANOVA compared with control. (D) Quantitative PCR and western blot (E) analysis of TCF7L2 overexpressed efficiency in GH3 cells (Student's *t*‐test, ****P  <  .0001, relative to control). (F,G) CCK‐8 assay was performed to detect the proliferative capacity of GH3 cells. *P*‐values by two‐way ANOVA, relative to control. (H,I) GH secretion in GH3 cells with knockdown and overexpression of TCF7L2 is shown. *P*‐values by one‐way ANOVA and Student's *t*‐test compared with control (**P  <  .01, ***P  <  .001). GH, Growth hormone.

Given the critical role of TCF7L2 in the Wnt signalling pathway, its structural and expressional dynamics in pituitary tumours are not well characterised. To elucidate these aspects, we examined the structural alternation and expression levels of TCF7L2 in pituitary tumours, aiming to understand its potential role and regulatory mechanisms in tumourigenesis. We observed that when TAD gained and loops were increased in TCF7L2 gene area, its expression was up‐regulated in tumour (Figure 4C). To verify whether the expression of TCF7L2 was increased in somatotroph tumours, qPCR was performed for 10 patients with somatotroph tumours and 3 normal pituitary glands from donators. Our results revealed that TCF7L2 was upregulated in somatotroph tumours (Figure 5A). To evaluate the role of TCF7L2 in tumourigenesis, we knocked down TCF7L2 using short hairpin RNA (shRNA) and overexpressed TCF7L2 using a cDNA clone plasmid. At both the mRNA and protein levels, TCF7L2 was efficiently depleted and upregulated, respectively, in GH3 cells (Figure 5B–E). The CCK‐8 assay showed that TCF7L2 knockdown inhibited cell proliferation, while TCF7L2 overexpression promoted cell proliferation (Figure 5F,G). In addition, ELISA revealed that downregulating TCF7L2 significantly reduced GH levels in GH3 cells, while upregulating TCF7L2 increased GH levels (Figure 5H,I). These data suggested that TCF7L2 promoted cell proliferation and GH secretion.

## 4 DISCUSSION

The structure of the genome in three dimensions plays a vital role in controlling several biological mechanisms, including DNA replication and the regulation of transcription.^3^, ^49^ However, the role of the 3D genome structure in somatotroph tumours has not been determined. Here, we first employed in situ Hi‐C to generate high‐resolution 3D genome maps and conducted RNA‐seq experiments to determine the changes in the 3D genome and how these changes related to the transcriptional responses in somatotroph tumour. We further investigated the functional and expressional changes of genes across various structural hierarchies. Notably, we focused on the structural alterations in TADs and loops, as well as the expression changes of TCF7L2, a critical gene in the Wnt signalling pathway. Further, we substantiated the functional role of TCF7L2 through in vitro experiments. This study enhances our comprehension of the elaborate molecular regulatory mechanisms implicated in the formation and progression of somatotroph tumours.

A comparison of Hi‐C profiles revealed that inter‐chromosomal associations between gene‐rich, small chromosomes (chr16–22) in somatotroph tumour was significantly reduced. Barutcu et al.^42^ proposed that one possible reason is the randomisation of contacts within the tumour genome, such as loss of specificity, which leads to the reduction of individual contact frequency and the obvious loss of interaction. Furthermore, the Hi‐C heatmap did not reveal abnormal inter‐chromosome interactions, which may be attributed to the generally benign nature of pituitary tumours. Extensive genomic studies have been conducted, yet pituitary tumour are characterised by comparatively fewer genetic aberrations than other types of tumour and cancer.^50^

Approximately 20% of the whole genome in somatotroph tumours has a switched compartment state compared to that in normal tissue. Consistent with previous findings,^4^ DEGs that converted from compartment B to A in somatotroph tumour were mostly downregulated, while DEGs that switched from compartment A to B exhibited significantly higher expression levels. Compartment A, associated with high transcriptional activity, is typically oriented towards the nucleus and genes. In contrast, compartment B, which adheres more closely to the nuclear membrane, is located near the nuclear periphery.^13^, ^51^ Additionally, we investigated the function of genes involved in AB switches and found that these genes are mainly enriched in the PI3K‐Akt and TGF‐beta signalling pathway. The activation of the PI3K‐Akt pathway in somatotroph tumours is well documented.^43^ Additionally, GSK3B, representing the mRNA of glycogen synthase kinase 3 beta (GSK‐3β), is a key element in the PI3K‐Akt pathway.^52^ We further investigated the set of genes undergoing A/B compartment switches within the PI3K‐Akt signalling pathway and found that the GSK3B gene region switched from compartment B in normal tissue to compartment A in somatotroph tumours. Additionally, its expression was upregulated in the somatotroph tumour, consistent with previous studies.^53^, ^54^

Based on the analysis of TAD boundaries, we found that tumour contained more TADs, with an average size smaller than that of normal tissue, indicating the presence of more newly formed TAD boundaries in the tumour. The emergence of new boundary can reduce the expression of tumour suppressors.^55^ In addition, we also observed changes in boundary strength in somatotroph tumours, which are disrupted by abnormal gene regulation or contribute to cancer.^10^ Specifically, function enrichment analysis indicated that these abnormal genes were predominantly concentrated in the Wnt‐beta Catenin signalling pathway, known to be activated in pituitary tumour.^56^, ^57^ Therefore, we investigated genes in the Wnt pathway associated with TAD changes and found that WNT7B, a key gene in the Wnt signalling pathway, showed enhanced insulation boundary and upregulated expression in tumours. Consist with our results, WNT7B is upregulated in several cancers, including prostate cancer^58^ and pancreatic cancer.^59^

Moreover, we identified chromatin loops by in situ Hi‐C and found that somatotroph tumour contained more loops than normal and that the average loop size was large. We further identified gained and lost loops in tumours that integrated these differences with DEGs to examine their functional relevance. Previous studies have reported that alterations in the loop structure, either by loss or gain, are influential in the regulation of gene expression and activation.^60^, ^61^ Furthermore, functional enrichment analysis indicated that genes associated with lost loops participate in the Wnt, Notch, and MAPK signalling pathways. The MAPK pathway is fundamentally important in role in cell proliferation and drug resistance in various human cancers,^62^ including pituitary tumours.^63^, ^64^ We investigated the genes associated with lost loops in the MAPK pathway and found that the NTF3 gene lost loops in somatotroph tumours and was downregulated in tumour. Yang et al^48^ reported that NTF3 was downregulated in hepatocellular carcinoma and that its low expression activates the MAPK pathway. In addition, genes associated with lost loops are also implicated in the Wnt signalling pathway, and TCF7L2 is a core component binding to nuclear β‐catenin to transduce Wnt signalling.^65^ Thus, we investigate the alternation in the genome architecture and gene expression of TCF7L2 and found that TADs gained and loops increased in the TCF7L2 gene area, with its expression being upregulated in the tumour. Based on previous literature reports,^8^, ^66^, ^67^ we hypothesise that TCF7L2 expression may be influenced by disruptions in TAD boundaries or chromatin loops, leading to dysregulated enhancer–promoter interactions. In addition, to explore the function of TCF7L2 in somatotroph tumour, we performed vitro experiments and found that TCF7L2 positively regulates tumour cell proliferation and GH secretion. Aligning with our findings, the research conducted by Xiang and colleagues demonstrated an upregulation of TCF7L2 in pancreatic cancer, which was associated with enhanced tumour cell proliferation.^68^ To conclusively determine whether mutations in genes associated with pituitary tumourigenesis might contribute to the altered genome topology observed in our tumours, we undertook whole‐genome sequencing at a depth of at least 100× for the tumour sample. Our analysis did not detect mutations in the genes GNAS, AIP, CDKN1B, and MEN1, supporting the conclusion that the changes in genome topology in our study samples are not directly linked to mutations in these known genes related to pituitary tumourigenesis (data not shown).

Although our study provides an initial insight in the 3D genomic landscape of somatotroph tumour, there are several limitations that need acknowledgement. First, due to the challenges in securing sufficient samples, the number of tumour and normal pituitary samples for Hi‐C experiments was modest. We chose typical somatotroph tumour samples and two normal pituitary tissue, reducing statistical errors by analysing multiple libraries. Additionally, all individuals contributing samples to our study are female, which introduces a potential gender bias into our findings. Therefore, future research must endeavor to include a broader and gender‐balanced sample range to enhance our understanding of 3D chromatin structural changes in the pituitary tumour. Furthermore, while we observed alterations in TADs and loops of TCF7L2 and preliminarily investigated its function through in vitro experiments, a comprehensive elucidation of its role in tumourigenesis requires further investigation. Despite these limitations, the current study has explored chromosomal changes at different hierarchical levels in tumours and normal pituitary and examined the relationship between these changes and gene expression, providing valuable insights into the mechanisms of pituitary tumour development and progression and potential therapeutic targets.

In summary, we presented a comprehensive analysis of genomic and transcriptomic alternations in somatotroph tumour, and revealed correlations between transcriptional regulation and 3D chromatin structural changes. This study sheds new light on the spatial aspects of somatotroph tumour development and progression, enhancing our understanding of their functional mechanisms and pathogenic processes.

## AUTHOR CONTRIBUTIONS

Yazhuo Zhang, Weiyan Xie and Chuzhong Li worked on the conception and designed research, and eventually approved the manuscript. Yiyuan Chen, Haibo Zhu, Xinyu Tong and Lei Cao contributed to collected and analysed clinical data of patients. Jing Guo performed the experiments, analysed the data, and wrote the manuscript. All authors read and approved the final manuscript.

## CONFLICT OF INTEREST STATEMENT

The authors declare no conflicts of interest.

## ETHICS APPROVAL

The study was approved by The Ethics Committees of Beijing Tiantan Hospital (KY2018‐053‐02). All subjects provided written informed consent.

## Supporting information

Supporting Information

Supporting Information

**FIGURE S1**. Correlation coefficients of eight Hi‐C libraries. The stratum‐adjusted correlation coefficient heatmap of Hi‐C libraries was calculated by Hicrep.

**FIGURE S2**. Hi‐C resolution depth of tumour (A) and normal pituitary tissue (B).

**FIGURE S3**. (A) *Cis*/*Trans* ratio for different libraries from tumour and normal pituitary tissues. (B) Proportions of *cis*‐interactions along each chromosome across various libraries from tumour and normal pituitary samples. (C) Box plot showing the *cis* ratio in each library from tumour and normal pituitary samples. (Student's *t*‐test, ***P  <  .001.)

**FIGURE S4**. (A,B) The circos plot showing the top 1000 inter‐chromosome interactions at 1‐Mb resolution in tumour (A) and normal pituitary tissue (B). The curve indicates the position of the 1 000 bin pairs with the strongest interactions. (C) The difference of top 1 000 inter‐chromosome interactions at 1‐Mb resolution between tumour and normal pituitary tissue. The red curve represents the unique bin pair of pituitary tumours, and the green curve represents the unique bin pair of normal pituitary gland.

**FIGURE S5**. Interaction decay exponents (IDEs) in tumour (A) and normal pituitary tissue (B). (C) Overall Hi‐C interaction frequency at different genomic distances for tumour and normal pituitary tissue.

**FIGURE S6**. The volcano plot showing the differentially expressed genes between tumour and normal pituitary tissue.

**FIGURE S7**. (A) Compartment A/B switching of whole chromosomes in tumour compared with normal pituitary. Assignment of the A compartment (deep red) and B compartment (deep blue) was performed using eigenvalues > 0 and < 0, respectively. (B) Comparison of A/B compartmental status of chromatins between tumour and normal pituitary. Each dot represents a 100 kb region.

**FIGURE S8**. (A) Box plots showing the comparison of gene expression levels in enhanced insulation boundary related genes and weak boundary related genes. (B) Volcano plot showing the DEGs at enhanced insulation boundaries. DEG, Differentially expressed gene.

## Data Availability

The datasets used and/or analysed during the current study are available from the corresponding author on reasonable request.
