# Supplementary material for: Three‐dimensional chromatin landscapes in somatotroph tumour
Source: Clin Transl Med. 2024 May 20;14(5):e1682. doi: 10.1002/ctm2.1682 (PMC11106515; doi:10.1002/ctm2.1682)
Supplement: Supplementary file 2 — Supporting Information [file CTM2-14-e1682-s005.docx]

**Supplementary Information for**

**Three-dimensional chromatin landscapes in somatotroph tumor**

Jing Guo^1,2^, Yiyuan Chen^1,2^, Haibo Zhu^1^, Xinyu Tong^3^, Lei Cao^1^, Yazhuo Zhang^2,4,5^, Weiyan Xie^2*^, Chuzhong Li^1,2,4,5*^

1 Department of Neurosurgery, Beijing Tiantan Hospital affiliated to Capital Medical University, Beijing 100070, China

2 Beijing Neurosurgical Institute, Capital Medical University, Beijing 100070, China

3 Annoroad Gene Technology Co., Ltd, Beijing, 100176, China.

4 Beijing Institute for Brain Disorders Brain Tumor Center, Beijing 100070, China

5 China National Clinical Research Center for Neurological Diseases, Beijing 100070, China

^*^**Corresponding author:**

E-mail: lichuzhong@ccmu.edu.cn, weiyanxie@ccmu.edu.cn

**Materials and Methods**

**Hi-C library preparation**

To prepare tissue samples for crosslinking, first remove the medium and introduce 22.5 ml of serum-free fresh medium per dish. Crosslink the tissues by adding 1.25 ml of 37% formaldehyde to achieve a 2% final concentration, mixing gently immediately after. Allow the tissues to incubate at room temperature for 10 minutes, gently rocking the dish every 2 minutes. Quench the crosslinking by adding 2.5 ml of 2.5 M glycine and mixing well, then incubate for 5 minutes at room temperature followed by at least 15 minutes on ice. Scrape the tissues from the dish and transfer them into a tube. After thorough mixing, divide the crosslinked tissue suspension into aliquots, each containing the equivalent of 25 x 10^6 cells. Centrifuge the aliquots at 800xg for 10 minutes, then carefully aspirate and discard the supernatant, ensuring complete removal of the liquid phase. At this point, the tissue can be snap-frozen in liquid nitrogen and stored at -80°C for up to 1.5 years, or one may proceed directly to tissue lysis.

The fixed tissue was resuspended in 1 ml of lysis buffer (10 Mm Tris-HCl pH 8.0, 10 mM NaCl, 0.2% Igepal CA-630, 1/10 vol. of proteinase inhibitor cocktail (Sigma)), and then incubated on ice for 20 minutes. Nuclei were pelleted by centrifugation at 4 °C, 600x g for 5 minutes, and then washed with 1 ml of the lysis buffer, followed by another centrifugation under similar conditions. After washing twice with restriction enzyme buffer, the nuclei were resuspended in 400 μl of restriction enzyme buffer and transferred to a safe-lock tube. Next, the chromatin is solubilized with dilute SDS and incubation at 65 ℃ for 10 min. After Quenching the SDS by Triton X-100 Overnight digestion was applied with 4 cutter restriction enzyme (400 units MboI) at 37℃ on rocking platform. The next steps are Hi-C specific, including marking the DNA ends with biotin-14-dCTP and performing blunt-end ligation of crosslinked fragments. The proximal chromatin DNA was religated by ligation enzyme. The nuclear complexes were reversed crosslinked by incubating with proteinase K at 65℃. DNA was purified by phenol-chloroform extraction. Biotin-C was removed from non-ligated fragment ends using T4 DNA polymerase. Fragments was sheared to a size of 200-600 base pairs by sonication. The fragment ends were repaired by the mixture of T4 DNA polymerase, T4 polynucleotide kinase and Klenow DNA polymerase. Biotin labeled HiC sample were specifically enriched using streptavidin C1 magnetic beads. The fragment ends were adding A-tailing by Klenow(exo-) and then adding Illumina paired-end sequencing adapter by ligation mix. At last, the Hi-C libraries were amplified by 12-14 cycles PCR, and sequenced in Illumina HiSeq platform. Sequencing interacting pattern was obtained by Illumina HiSeq instrument with 2×150-bp reads.

**RNA sequencing**

For the RNA quality examination, we assessed the purity of our samples with the kaiaoK5500® Spectrophotometer and evaluated RNA integrity and concentration using the RNA Nano 6000 Assay Kit of the Agilent Bioanalyzer 2100 system.

In the preparation of RNA sequencing libraries, we started with 2 μg of RNA per sample. The NEBNext® Ultra™ RNA Library Prep Kit for Illumina® was used according to the manufacturer's protocol, which included adding index codes to each sample for sequence attribution. The process involved purifying mRNA from the total RNA using poly-T oligo-attached magnetic beads, followed by fragmentation using divalent cations at an elevated temperature. The cDNA was synthesized in two stages: the first strand using a random hexamer primer and RNase H, and the second strand using a specialized buffer, dNTPs, DNA polymerase I, and RNase H. After purification of the library fragments with QiaQuick PCR kits and elution with EB buffer, we performed terminal repair, A-tailing, and adapter ligation. The final step was PCR amplification to complete the library construction.

The RNA sequencing library construction involved concentrating mRNA, fragmenting it, synthesizing cDNA, and then performing end repair. This was followed by A-tailing, adapter ligation, and selection of the appropriate fragment size. Finally, the library was purified. Key reagents included the NEBNext super speed RNA Library Prep Kit for Illumina® and Beckman AM Pure XP beads, with ethanol for purification and PCR instruments for amplification.RNA concentration of library was measured using Qubit® RNA Assay Kit in Qubit® 3.0 to preliminary quantify and then dilute to 1ng/μl. Insert size was assessed using the Agilent Bioanalyzer 2100 system (Agilent Technologies, CA, USA), and qualified insert size was accurate quantification using StepOnePlus™ Real-Time PCR System (Library valid concentration＞10 nM).The clustering of the index-coded samples was performed on a cBot cluster generation system using HiSeq PE Cluster Kit v4-cBot-HS (Illumina) according to the manufacturer’s instructions. After cluster generation, the libraries were sequenced on an Illumina platform and 150 bp paired-end reads were generated.
