# Supplementary figures and images for: Three‐dimensional chromatin landscapes in somatotroph tumour
Source: Clin Transl Med. 2024 May 20;14(5):e1682. doi: 10.1002/ctm2.1682 (PMC11106515; doi:10.1002/ctm2.1682)

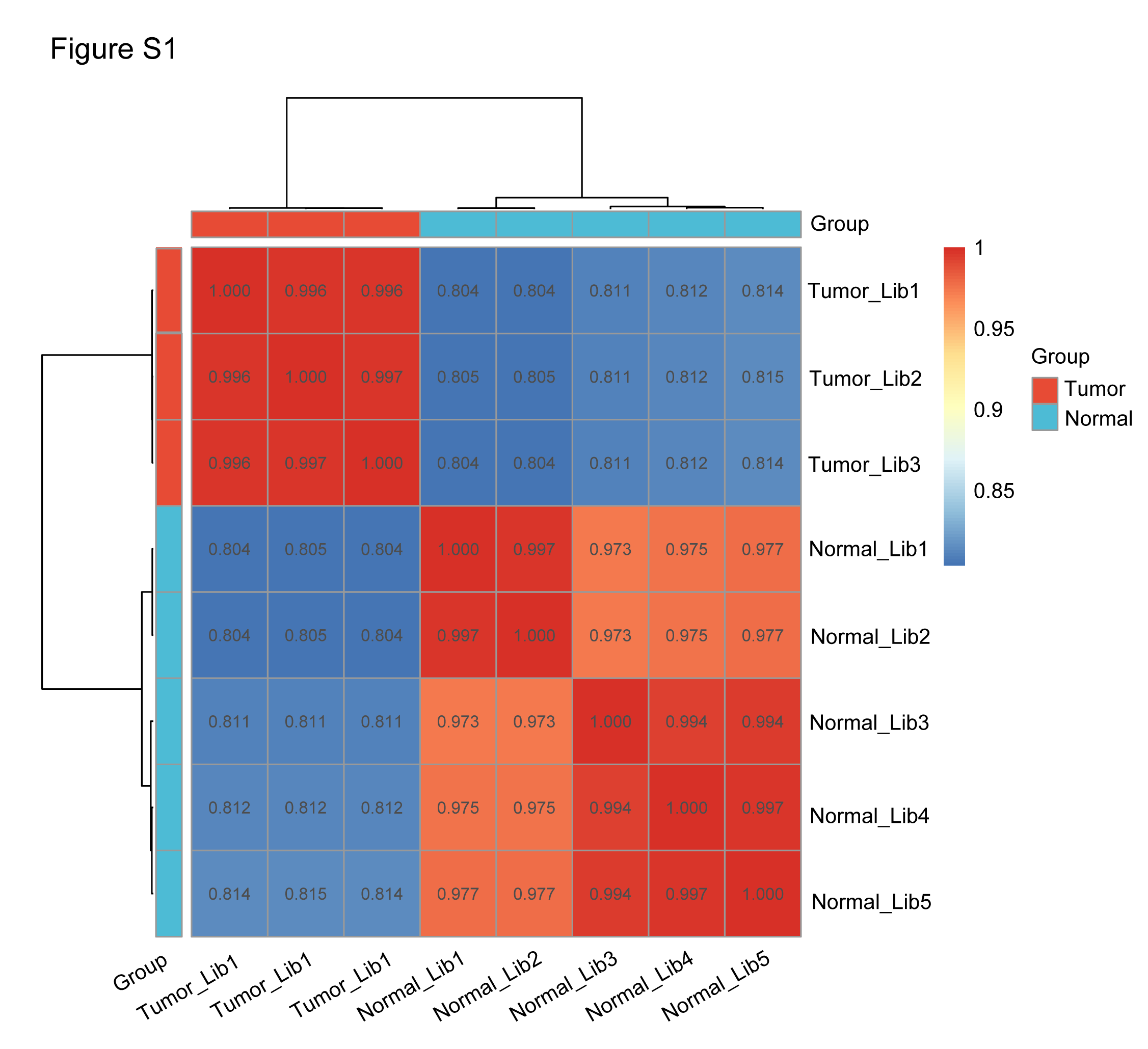

Supplement: Supplementary file 3 — FIGURE S1. Correlation coefficients of eight Hi‐C libraries. The stratum‐adjusted correlation coefficient heatmap of Hi‐C libraries was calculated by Hicrep. [file CTM2-14-e1682-s002.tif]

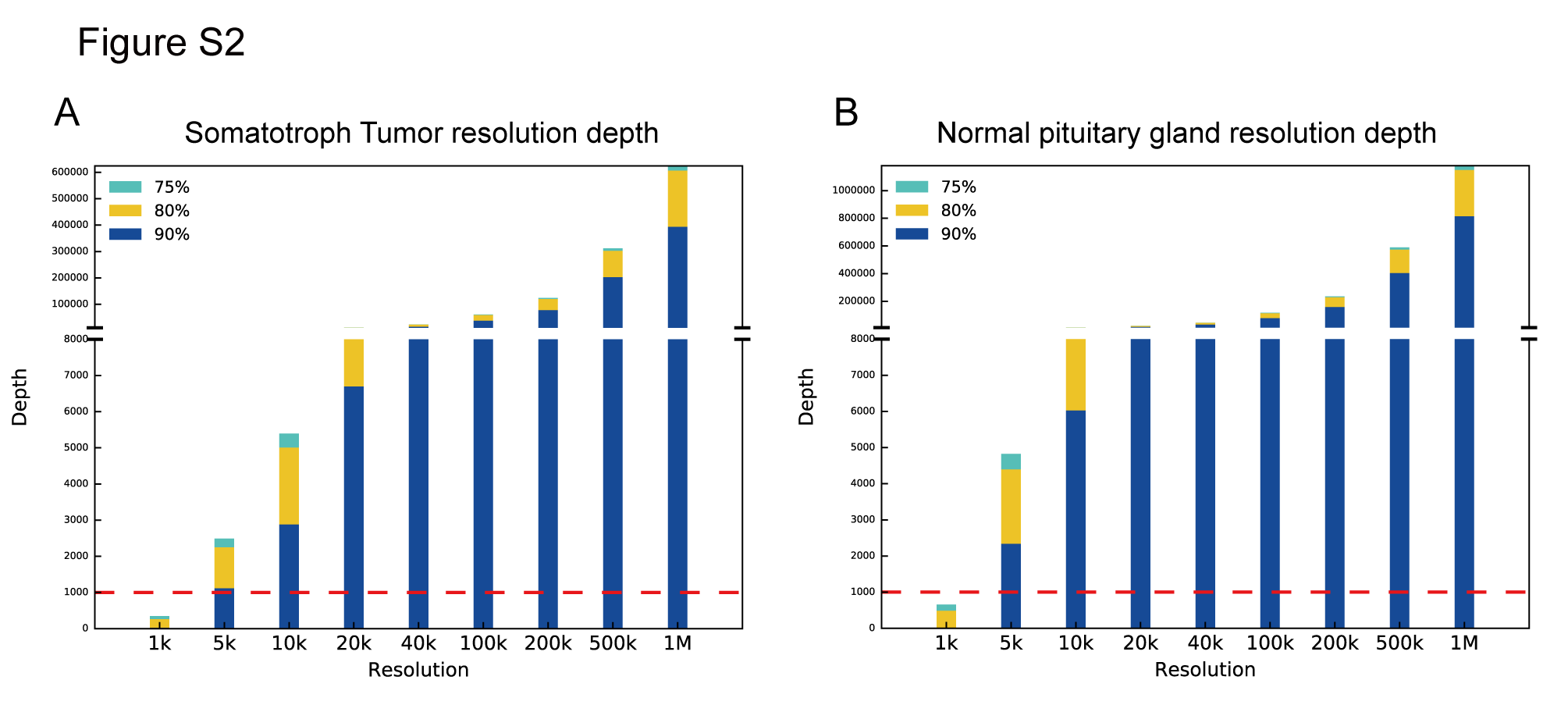

Supplement: Supplementary file 4 — FIGURE S2. Hi‐C resolution depth of tumour (A) and normal pituitary tissue (B). [file CTM2-14-e1682-s001.tif]

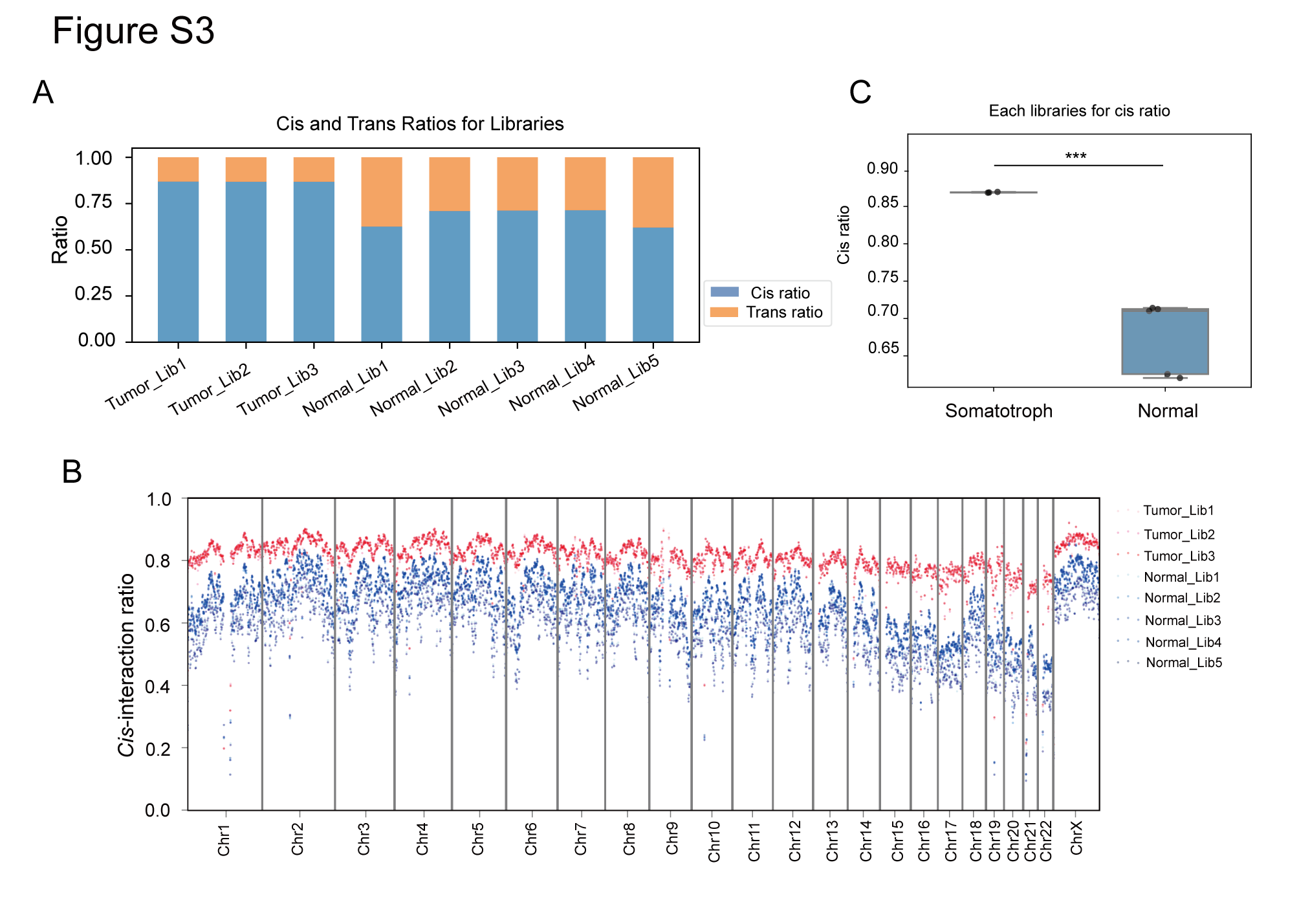

Supplement: Supplementary file 5 — FIGURE S3. (A) Cis/Trans ratio for different libraries from tumour and normal pituitary tissues. (B) Proportions of cis‐interactions along each chromosome across various libraries from tumour and normal pituitary samples. (C) Box plot showing the cis ratio in each library from tumour and normal pituitary samples. (Student's t‐test, ***P < .001.) [file CTM2-14-e1682-s006.tif]

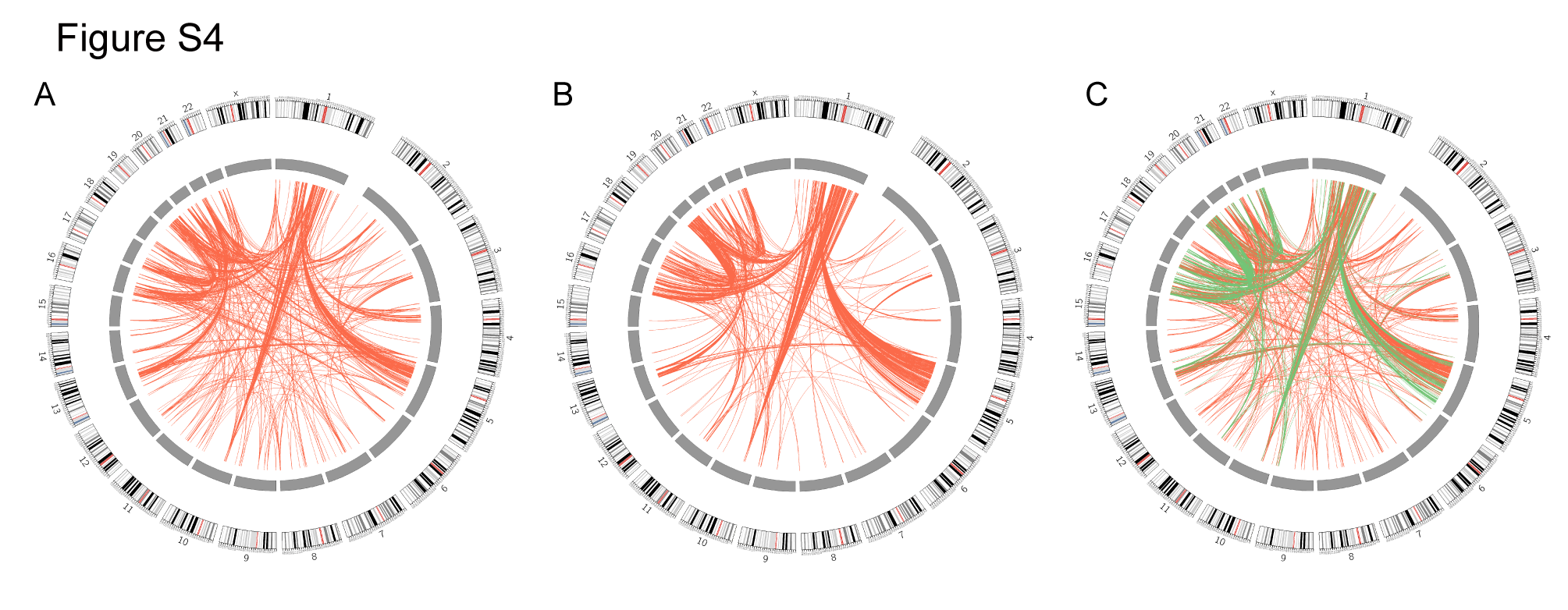

Supplement: Supplementary file 6 — FIGURE S4. (A,B) The circos plot showing the top 1000 inter‐chromosome interactions at 1‐Mb resolution in tumour (A) and normal pituitary tissue (B). The curve indicates the position of the 1 000 bin pairs with the strongest interactions. (C) The difference of top 1 000 inter‐chromosome interactions at 1‐Mb resolution between tumour and normal pituitary tissue. The red curve represents the unique bin pair of pituitary tumours, and the green curve represents the unique bin pair of normal pituitary gland. [file CTM2-14-e1682-s009.tif]

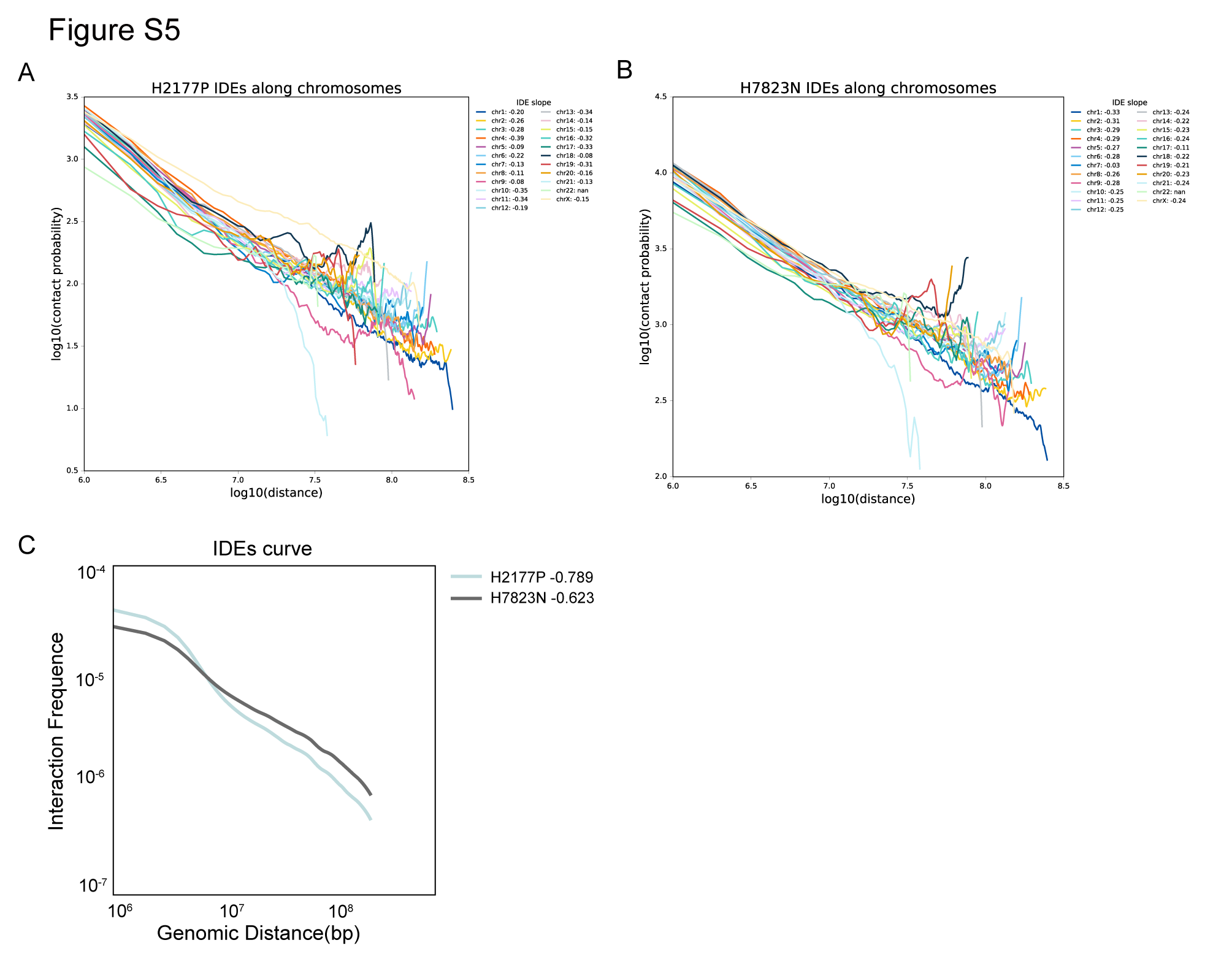

Supplement: Supplementary file 7 — FIGURE S5. Interaction decay exponents (IDEs) in tumour (A) and normal pituitary tissue (B). (C) Overall Hi‐C interaction frequency at different genomic distances for tumour and normal pituitary tissue. [file CTM2-14-e1682-s003.tif]

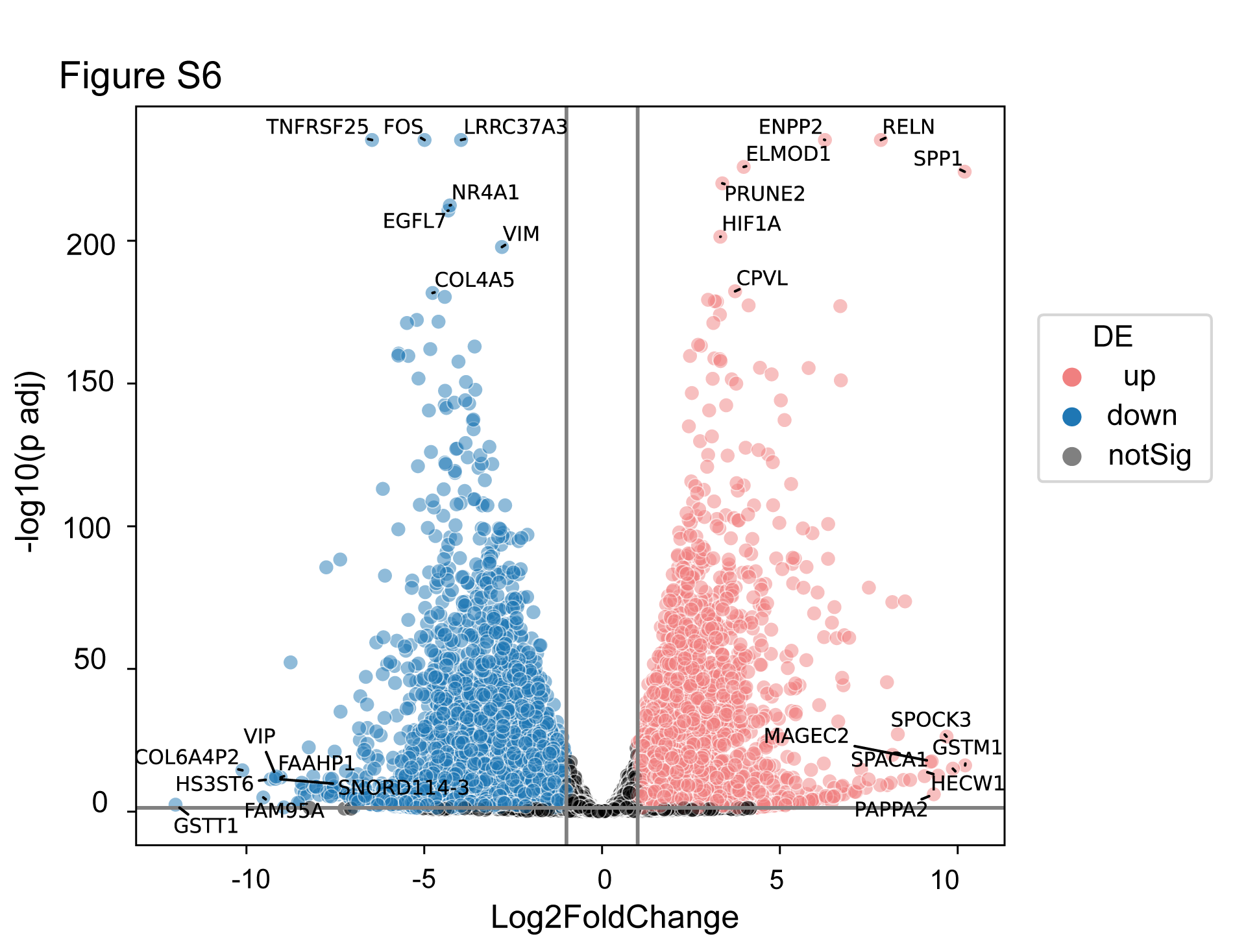

Supplement: Supplementary file 8 — FIGURE S6. The volcano plot showing the differentially expressed genes between tumour and normal pituitary tissue. [file CTM2-14-e1682-s007.tif]

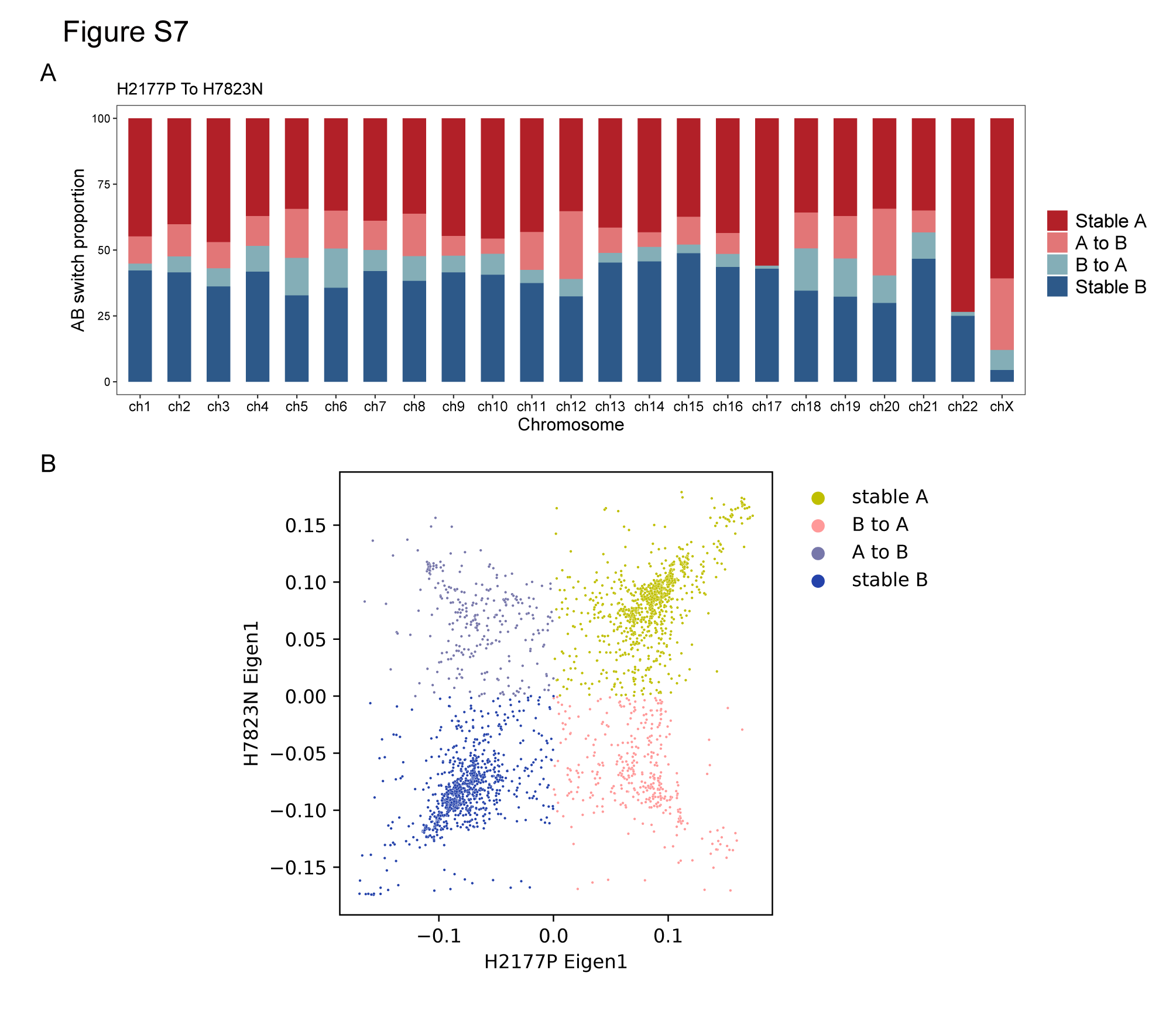

Supplement: Supplementary file 9 — FIGURE S7. (A) Compartment A/B switching of whole chromosomes in tumour compared with normal pituitary. Assignment of the A compartment (deep red) and B compartment (deep blue) was performed using eigenvalues > 0 and < 0, respectively. (B) Comparison of A/B compartmental status of chromatins between tumour and normal pituitary. Each dot represents a 100 kb region. [file CTM2-14-e1682-s008.tif]

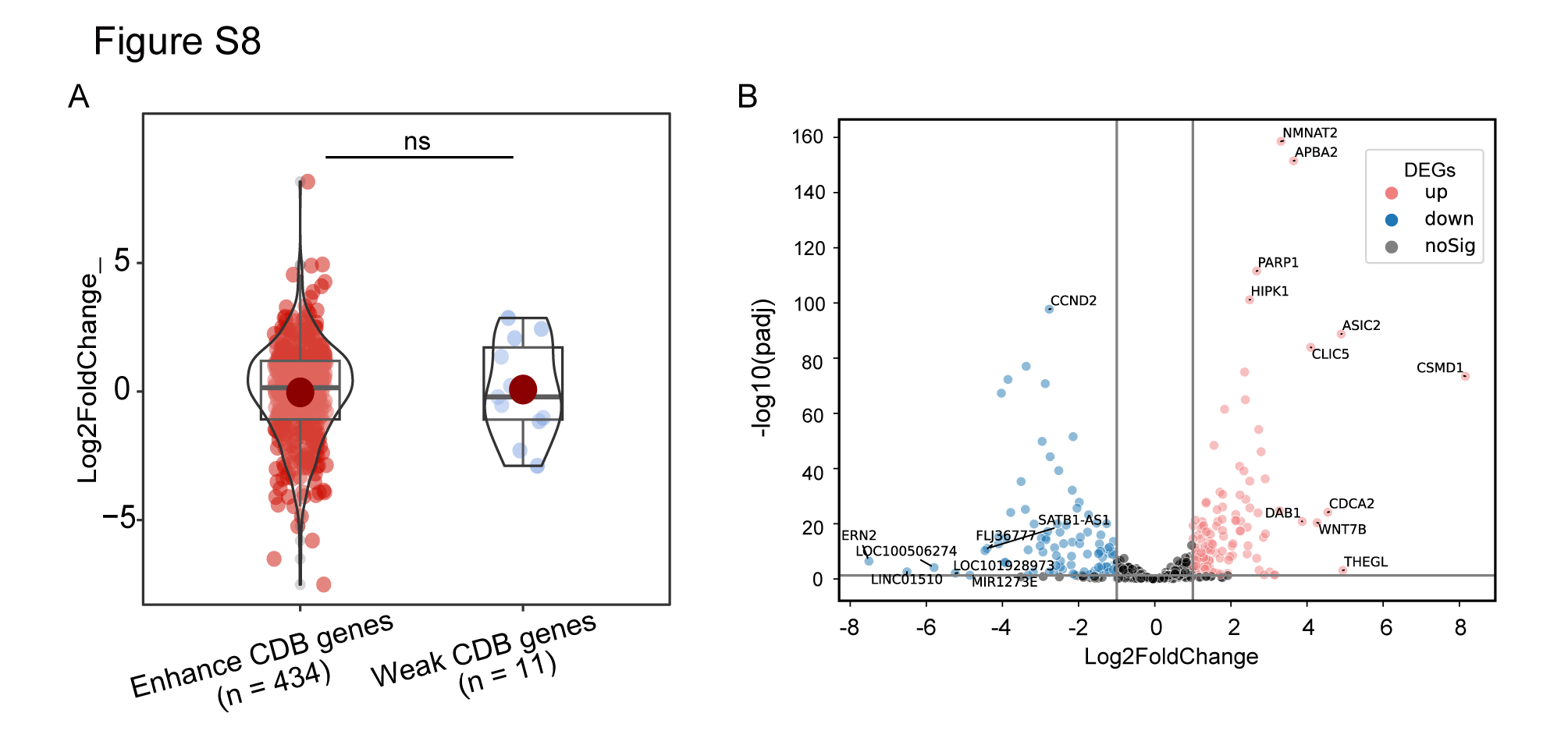

Supplement: Supplementary file 10 — FIGURE S8. (A) Box plots showing the comparison of gene expression levels in enhanced insulation boundary related genes and weak boundary related genes. (B) Volcano plot showing the DEGs at enhanced insulation boundaries. DEG, Differentially expressed gene. [file CTM2-14-e1682-s010.tif]
